# Advantages of targeting the tumor immune microenvironment over blocking immune checkpoint in cancer immunotherapy

**DOI:** 10.1038/s41392-020-00449-4

**Published:** 2021-02-20

**Authors:** Tianyu Tang, Xing Huang, Gang Zhang, Zhengtao Hong, Xueli Bai, Tingbo Liang

**Affiliations:** 1 Zhejiang Provincial Key Laboratory of Pancreatic Disease, The First Affiliated Hospital, School of Medicine, Zhejiang University, 310003 Hangzhou, Zhejiang China; 2 Department of Hepatobiliary and Pancreatic Surgery, The First Affiliated Hospital, School of Medicine, Zhejiang University, 310003 Hangzhou, Zhejiang China; 3 Innovation Center for the Study of Pancreatic Diseases, Zhejiang Province, 310003 Hangzhou, Zhejiang China; 4 Zhejiang University Cancer Center, 310003 Hangzhou, Zhejiang China

**Keywords:** Cancer microenvironment, Tumour immunology

## Abstract

Despite great success in cancer immunotherapy, immune checkpoint-targeting drugs are not the most popular weapon in the armory of cancer therapy. Accumulating evidence suggests that the tumor immune microenvironment plays a critical role in anti-cancer immunity, which may result in immune checkpoint blockade therapy being ineffective, in addition to other novel immunotherapies in cancer patients. In the present review, we discuss the deficiencies of current cancer immunotherapies. More importantly, we highlight the critical role of tumor immune microenvironment regulators in tumor immune surveillance, immunological evasion, and the potential for their further translation into clinical practice. Based on their general targetability in clinical therapy, we believe that tumor immune microenvironment regulators are promising cancer immunotherapeutic targets. Targeting the tumor immune microenvironment, alone or in combination with immune checkpoint-targeting drugs, might benefit cancer patients in the future.

## Introduction

To date, immune checkpoint (ICP)-targeting drugs, such as anti-cytotoxic T-lymphocyte-associated protein 4 (CTLA-4), anti-programmed cell death protein 1 (PD-1), and anti-PD-1 ligand 1 (PD-L1), have displayed considerable success in a number of cancer immunotherapies,^1–4^ including melanoma, lung cancer, and other commonly diagnosed cancers. Accordingly, ICP blockade-based therapeutic strategies have been championed in cancer research and therapy, often in the name of patient benefit. Currently, for patients with advanced head and neck squamous cell carcinoma, non-small cell lung cancer (NSCLC, squamous and non-squamous carcinoma), melanoma, urothelial and kidney cancers, Merkel cell carcinoma, refractory Hodgkin lymphoma, microsatellite instability-high colorectal cancer, gastric cancer, and hepatocellular carcinoma, therapeutic ICP blockade has become a part of the standard of care. Clinical trials have been initiated to investigate their efficacy for the treatment of additional malignant diseases.^5–9^ However, increasing numbers of studies have shown that the positive response rate among patients receiving immune checkpoint-targeting drugs remains quite low, an issue that remains to be solved.^10,11^ Previous studies have demonstrated that in the majority of the cases, clinical benefit is commonly prevented by acquired resistance to the tumor and primary tumor refractoriness to the ICP-targeting drugs.^12^ Furthermore, clinical decisions to use these drugs, especially dual CTLA-4 and PD-1 blockade, should consider their potential to induce high-grade immune-related adverse events. Thus, the clinical practice of checkpoint-targeting therapy remains problematic.

Increasing evidence strongly suggests that the tumor immune microenvironment (TIME) plays a more significant role than ICPs in tumor immune surveillance and immunological evasion.^13–15^ Many multiple factors co-contribute to anti-cancer immunity, and ICPs are just one weapon used by tumors to resist attack from the immune system.^16^ Therefore, further improvements are required for therapeutic precision to limit the side-effects of therapies that are based on targeting ICPs, in addition to other cancer therapies that are both relevant or not. In the present review, we aim to provide new insights into current cancer immunotherapy and reveal potential antitumor immunological targets in the TIME that overcome immunotherapeutic resistance in clinical applications. We highlight the significance and superiority of targeting TIME regulators in anti-cancer immunotherapy. Furthermore, we discuss the potential and feasibility of combined treatments to boost a controllable anti-cancer immune response.

## Lessons learned from immune checkpoint-targeting cancer therapy

Drugs that target ICPs, including but not limited to anti-CTLA-4 and anti-PD-1/PD-L1, significantly improve the prognosis of advanced cancer patients. However, an increasing number of recent reports have provided contrary but convincing evidence of non-negligible defects in ICP-targeting strategies, which might have negative consequences for their therapeutic efficacy.^17–22^ Generally, the efficacy of ICP therapy is restricted by three major factors: (1) burden of tumor mutation, (2) PD-L1 expression level, and (3) pre-existing T-cell infiltration. In fact, with the exception of melanoma,^23^ Merkel cell carcinoma,^24^ and Hodgkin disease,^25^ the response rate to ICP monotherapy remains low in several specific malignancies, including pancreatic cancer, cholangiocarcinoma, and gastric cancer.^26–28^ In these cases, the objective response rate ranges from 15 to 25%, with limited survival benefit.^6,29–34^ In addition, concerns over safety-related problems have arisen in multiple cancer therapies, restricting the widespread use of ICP blockade.^35,36^ ICPs not only affect cross-talk between the immune system and a tumor, acting as a gatekeeper toward anti-cancer immunity, they also function within the immune system, serving as a vital mechanism to maintain immunological homeostasis.^35^ Consequently, the toxicity of ICP-targeting drugs differs from that of conventional chemotherapies, targeted small molecule inhibitors, or even traditional therapeutic monoclonal antibodies.^36^ Non-specific activation of immune responses via ICP blockade can lead to immune-related adverse events (irAEs).^36–40^ The incidence of all grades of irAE is reported to range from 15 to 90%, and the frequency of severe irAEs requiring immunosuppression and withdrawal from immunotherapy is estimated to be between 0.5 and 13%.^41^ Disordered infiltration of immune cells in normal skin, and gastrointestinal, hepatic, thyroid, renal, pulmonary, musculoskeletal, and pituitary tissues has been reported in cancer patients receiving ICP-targeted therapies.^42,43^ These irAEs can lead to treatment interruption and even multiple organ failure. Unfortunately, the pathophysiology of irAEs resulting from ICP blockade is not yet fully understood. Even using dosage-decreasing countermeasures, complete prevention of such toxicity is unachievable in the short-term, which represents a significant challenge to ICP-centric immunotherapy. Thus, ICP-targeting inhibition should be precisely controlled to avoid potentially severe autoimmune disorders or infectious diseases in clinical practice.

## Multiple immunosuppressive tumor microenvironments dominate cancer immunotherapeutic efficacy

**Fig. 1:**
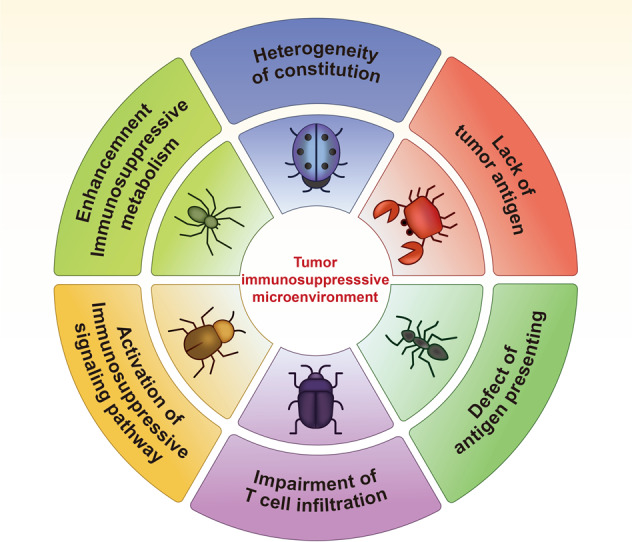
Hallmarks of an immunosuppressive tumor microenvironment. Six hallmarks, including heterogeneity of constitution, lack of tumor antigen, defect of antigen-presenting cell, impairment of T-cell infiltration, activation of an immunosuppressive signaling pathway, and enhancement of immunosuppressive metabolism co-contribute to an immunosuppressive tumor microenvironment

As described earlier, in addition to ICPs, TIME largely determines the therapeutic efficacy of cancer immunological treatments. Several hallmarks of immunosuppressive tumor microenvironment and their influence on current cancer immunotherapy can be summarized as follows (Fig. 1).

### Heterogeneity of constitution

**Fig. 2:**
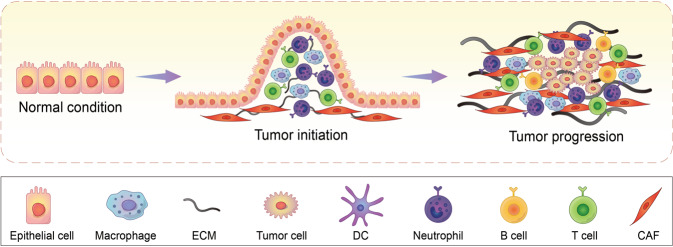
Construction of TIME. During tumorigenesis and progression, a variety of cells, including but not limited to macrophage, DC, neutrophil, B cell, T cell, and CAF, are recruited to the surrounding microenvironment of tumor cells, co-constituting the TIME together with the ECM in addition to other elements

It has been demonstrated that a variety of cells are recruited to the TIME during the development of a tumor. The composition of the heterogeneous TIME is extremely complex, containing a variety of immunosuppressive cells, including tumor cells, cancer-associated fibroblasts (CAFs), vascular endothelial cells, suppressive myeloid cells, regulatory T (Treg) cells, and regulatory B cells (Fig. 2). Each cell type in a suppressive TIME contributes to its promotion and maintenance. The immunoregulatory potential of stromal cells has recently received increased attention. These cells have been demonstrated to suppress the immune system by inhibiting the trafficking and function of T cells by both direct and indirect mechanisms.^44^ Furthermore, as will be discussed, the molecular deregulation in endothelial cells also plays a critical role in the inhibition of T-cell trafficking and induction of T-cell apoptosis. Myeloid subsets, including tumor-associated macrophages, monocytes and granulocytes, constitute the heterogeneous components of the TIME and have strong immunosuppressive potential.^45^ These cells inhibit the antitumor activity of T cells and natural killer (NK) cells using a variety of mechanisms, leading to resistance to immunotherapy.^46,47^ Mounting evidence demonstrates that the recruitment and activation of myeloid subsets are associated with tumor progression, recurrence, and negative clinical outcome. Treg cells play a critical role in the maintenance of immune homeostasis by inhibiting abnormal/excessive immune responses. However, previous studies have demonstrated that Treg cells promote tumor development and progression by inhibiting antitumor immunity. The underlying suppressive mechanisms include: inhibition of costimulatory signals mediated via CD80 and CD86 expressed by dendritic cells, secretion of inhibitory cytokines, metabolic modulation of tryptophan and adenosine, and direct killing of effector T cells.^48^ Regulatory B cells were also found to be involved in the development and maintenance of immunological tolerance by producing chemokines such as IL-10, IL-35, and transforming growth factor β (TGFβ). Considering the heterogeneity of the TIME, application of ICP inhibitors is perhaps not sufficient to maximize the benefit of immunotherapy, and the use of tumor biomarkers involved in the maintenance of an immunosuppressive microenvironment should also be considered to achieve better outcomes and safety. In a proportion of patients at least, the observed response of ICP inhibitors may result from the capacity of these therapies to simultaneously reshape the TIME. However, rather than identifying immunotherapies as ineffective, the observed resistance may indicate that both the influence and stimulation of factors in the TIME cannot meet the minimum requirements for reinvigorating the immune system. Therefore, it is timely to incorporate ICP inhibitors into more effective combination therapies.

### Lack of tumor antigen

There are three principal categories of tumor antigen: tumor-specific antigens, tumor germline antigens and tumor-associated antigens. For the majority of malignancies without viral etiology, tumor-specific antigens are formed solely by tumor-specific gene mutations. Cancer germline antigens are expressed in tumor tissues but silent in most normal tissues except in trophoblastic and male germline cells. Tumor-associated antigens are normally expressed at low levels in normal tissues, but at high levels in tumor cells. These antigens are important for the differentiation of cancer cells from normal cells by the immune system. However, compared with the non-mutated self-antigens with incomplete T-cell tolerance, mutated neoantigens are believed to be significantly more relevant to antitumor immune function. Tumor mutational load is used to quantify the number of mutations per coding region of the tumor genome. It is reasonable to believe that tumors with a high mutational load tend to express a greater number of tumor-specific antigens. Previous studies have shown that a high tumor mutational load is associated with a favorable immune response in patients who received ICP therapy. By analyzing the mutation profiles of 336 patients who received ICP blockade therapy, Chen et al. demonstrated that high neoantigen quality was associated with prolonged survival when receiving immunotherapy (log-rank test, *P* = 0.009).^49^ Eliezer et al. reported that nonsynonymous mutational load was significantly associated with the clinical benefit of ipilimumab therapy for melanoma (*P* = 0.0076; Mann–Whitney test).^50^ In patients with NSCLC, Rizvi reported that patients with a low nonsynonymous mutation burden suffered a lower objective response rate and shorter progression-free survival.^51^ These observations confirmed the hypothesis that neoantigens, caused by nonsynonymous mutations, are critical for the tumor response to ICP therapy. However, a previous report indicated that cancer cells develop particular mechanisms to avoid being eliminated after recognition by the immune system, which is termed immune cancer immunoediting. These mechanisms include reducing the expression of the most immunogenic antigen and loss of the mutation that results in an immunogenic neoantigen.^52^ In tumors with a low mutational load, the lack of sufficient neoantigens could lead to a state of low immunogenicity, which would eventually result in T-cell exclusion. In addition, cancer antigens can undergo direct modification such as glycosylation or cleavage by extracellular matrix metalloproteinases to avoid recognition by the immune system.^53–56^ These events can cause both primary and acquired resistance to ICP therapy in different cancers, ultimately resulting in unsuccessful treatment.

### Defect of antigen-presenting cell

While direct presentation of antigens by tumor cells in the context of MHC-I molecules plays an important role in the activation of an antitumor immune response, cross-presentation by professional antigen-presenting cells (APCs), especially dendritic cells (DCs), is the foundation of the “cancer immunity cycle.” DCs have been identified as remarkably capable cytotoxic T-cell stimulators that prolong the survival of cancer patients.^57,58^ Roberts et al.^59^ reported that CD103^+^ DCs are the dominant cell type responsible for tumor antigen cross-presentation. All such effects require CCR7 and high CCR7 expression levels in melanoma were found to be significantly related to T-cell infiltration and better clinical outcomes in patients. Spranger et al.^60^ investigated the underlying mechanism of immune resistance in tumors using a tumor model resembling cold tumors (lack of tumor T-cell infiltration) in humans. Their results indicate that tumor infiltrating CD103^+^ DCs played an important role in T-cell trafficking by producing C-X-C motif chemokine ligand 9/10 (CXCL9/10). However, the tumor-mediated suppressive microenvironment modulates DCs and suppresses their ability through a variety of mechanisms that eventually lead to tumor immune escape. A variety of TIME factors have demonstrated to negatively impact DCs.^61^ Tumor-derived interleukin-6 (IL-6) and macrophage colony-stimulating factor were found to be responsible for switching the differentiation of CD34^+^ progenitors from DCs to monocytes, which lack APC function.^62^ Tumor-derived IL-10 has also been shown to be responsible for DC dysfunction by inhibition of DC maturation that impairs their antigen presentation capacity.^63^ Other factors, including matrix metalloproteinase 2 and thymic stromal lymphopoietin were found to skew T-cell differentiation by modulating DC function.^64,65^ The β-catenin signaling pathway has also been shown to be responsible for DC dysfunction. The tumor-derived Wnt5a ligand significantly increases the expression and activity of indoleamine 2,3-dioxygenase-1 (IDO1) in DCs via the β-catenin pathway, leading to promotion of Treg differentiation.^66^ Hong et al.^67^ reported that DC-specific deletion of β-catenin in mice markedly enhanced antitumor immune response and delayed tumor growth. Considering all these mechanisms may contribute to primary and acquired resistance to immunotherapy as insufficient antigen presentation may lead to T-cell anergy and restriction of antitumor immunity, it is logical to combine DC-therapy and ICBs to achieve synergistic effects and improve the clinical response.

### Impairment of T-cell infiltration

Tumor infiltrating CD8^+^ T cells play a critical role in the response to immunotherapy. It was hypothesized that the activity of ICP therapy relied mostly on pre-existing CD8^+^ T-cell infiltration.^68^ For example, Tumeh et al.^69^ investigated 46 patients with advanced melanoma who received anti-PD-1 therapy. Pretreatment samples obtained from the patients displayed a higher CD8^+^ cell density at the invasive tumor margin in patients who experienced a tumor response. In an investigation of anti-PD-1/ anti-PD-L1 therapy in 32 patients with melanoma, Eroglu et al.^23^ reported a higher baseline T-cell density in patients with prolonged survival (*P* = 0.002). However, T-cell infiltration of tumors can be inhibited by a variety of immunosuppressive mechanisms. Firstly, T-cell trafficking can be influenced by the heterogeneity of perfusion and oxygen levels across the regions of the tumor caused by abnormalities in tumor neovasculature. In addition, the adhesion process can also be modulated in tumors to exclude antitumor T cells. The downregulation of intercellular adhesion molecule 1/2, vascular cell adhesion molecule 1 and overexpression of the endothelin B receptor on endothelial cells were also shown to be associated with the absence of infiltrating T cells.^70–72^ In ovarian cancer, silencing of *CXCL9* and *CXCL10* expression in tumor cells via an epigenetic mechanism inhibited T-cell infiltration.^73^ Furthermore, further evidence of the involvement of pro-inflammatory chemokines in the inhibition of T-cell infiltration was provided by the observation that intratumoral reactive nitrogen species produced by suppressive myeloid cells were able to induce CCL2 chemokine nitration and hinder T-cell migration and infiltration. Moreover, tumors rely on other components for T-cell exhaustion. For example, the tumor stroma, including CAFs, can suppress T-cell activation and inhibit T-cell infiltration. Furthermore, an in vivo model indicated that the reduction of collagen enhanced T-cell infiltration, suggesting that the density of extracellular matrix is associated with the ability of T cells to migrate.^74^

### Activation of immunosuppressive signaling pathway

Tumor cells are associated with molecular alterations, including, but not limited to, mutations of Kirsten rat sarcoma viral oncogene (Kras), focal adhesion kinase (FAK), and Janus kinase 1/2 (JAK1/2), which directly influence the TIME and immune function. As a signature event of tumor development, Kras mutations are common in a variety of malignancies and play an important role in the development and maintenance of the TIME, in addition to controlling tumor metabolism.^75^ Tumor cells with Kras mutations in vivo were found to induce granulocyte-monocyte colony-stimulating factor, promoting the accumulation of suppressive myeloid cells and Treg cells, leading to T-cell exhaustion.^76^ In a Kras-driven mouse model, Kras^G12D^ upregulated Hedgehog signaling and activated the inflammatory pathway in autochthonous pancreatic tumors, which promoted the development and maintenance of a fibroinflammatory stroma resulting in T-cell exhaustion.^77^ In addition, FAK was also identified as a critical regulator of the TIME. In squamous cell carcinoma, nuclear FAK promotes tumor growth and T-cell exhaustion by inducing CCL5 to recruit Treg cells.^78^ FAK has also been shown to negatively regulate T-cell receptor-mediated signaling by influencing the recruitment of C-terminal Src kinase members following TCR activation in T cells.^79^ Preclinical data have shown that the FAK inhibitor VS-4718 reduced tumor fibrosis, decreased the number of myeloid-derived suppressor cells and prolonged survival in a mouse model.^80^ Moreover, when experiencing immune attack, cytotoxic T lymphocytes release interferon gamma (IFNγ) into the TIME, which further activates signal transducers and activators of transcription-related signaling pathways in cancer cells, in turn upregulating PD-L1 expression that suppresses the immune attack.^81–84^ Acquired PD-1 blockade resistance in melanoma was found to correlate with JAK1 and JAK2 loss-of-function mutations. Such mutations blocked IFNγ signaling, resulting in insensitivity to its antiproliferative effects on cancer cells.^85^ These altered signaling pathways play a critical role in maintaining an immunosuppressive microenvironment, presenting a major obstacle for cancer immunotherapy.

### Enhancement of immunosuppressive metabolism

**Fig. 3:**
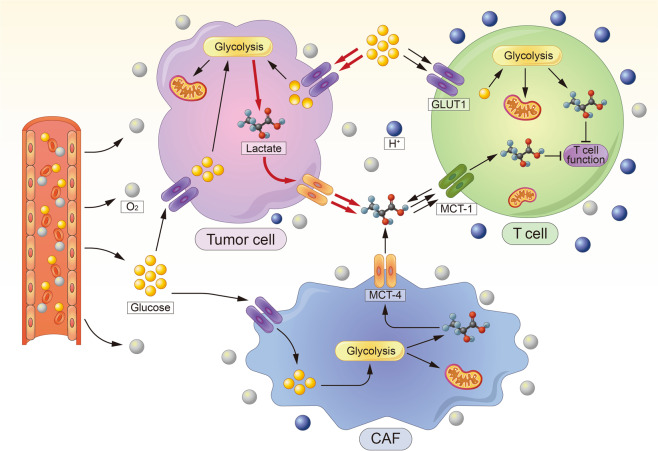
Metabolic regulation of tumor microenvironment for T-cell energy. The high rate of aerobic glycolysis in tumor cells and CAFs deprives immune cells of nutrients that are critical for their physiological function, meanwhile causing increased lactate production, which leads to tumor microenvironment acidification and immunosuppression

The desmoplastic response and elevated energy production rate of tumors create a hypoxic and low-nutrient extracellular environment, which is unfavorable for the survival of both tumor cells and immune cells. However, compared with immune cells, tumor cells have extraordinary metabolic plasticity, which facilitates their adaptation to and survival in harsh conditions, further depriving immune cells of nutrients critical for proliferation and function (Fig. 3). It has been demonstrated that metabolic alterations play an important role in the maintenance of an immunosuppressive environment.^86^ Glucose is a predominant fuel source in proliferating cells. The Warburg effect, which represents the metabolic switch from cellular respiration to anaerobic glycolysis, is a hallmark of tumor metabolism. Previous studies have shown that tumors undergo metabolic reprogramming to compete for this vital, but limited nutrient source. The hypoxic conditions and oncogenic molecules in tumor cells cause upregulation of the expression of the receptor for glucose internalization, GLUT1, in addition to other genes of metabolism.^87,88^ As a result, tumors undergoing immune reprogramming with increased import-receptor expression outcompete immune cells for glucose. Previous studies have shown that GLUT1 overexpression was associated with T-cell exhaustion in a variety of malignancies.^89,90^ T cells also undergo metabolic switching to adapt to conditions of limited glucose availability. Interestingly, while Glut1 deficiency was found to impair effector T-cell expansion and function, Treg cells, in contrast, appeared functionally unaffected in vivo.^91^ However, Glut1 deficiency impairs the antitumor response of CD8^+^ T cells. GLUT1-lo T cells exhibited decreased effector phenotype acquisition, reduced proliferation, and impaired infiltration within both hypoxic and normoxic conditions.^92^ High rates of aerobic glycolysis also cause increased lactate production, which leads to tumor microenvironment acidification. Lactate production is associated with metastasis, angiogenesis and immunosuppression. Excessive lactate induces NK cell apoptosis by decreasing the intracellular pH, resulting in NK cell depletion in colorectal liver metastasis.^93^ Lactic acid also suppresses the proliferation of CTLs and impairs their cytokine production capability, which is recovered in lactic acid-free medium.^94^ The growth of tumors with reduced lactate production is substantially slower than that of control tumors and is accompanied by increased infiltration of IFNγ-producing T and NK cells in vivo.^95^ Elevated catabolism of the amino acids tryptophan and arginine is an additional hallmark of the TIME in a variety of malignancies.^96^ IDO, tryptophan 2,3-dioxygenase (TDO) and arginase play essential roles in tryptophan and arginine catabolism and regulate T-cell immunity in an inflamed TIME.^97–100^ The metabolism of tryptophan and arginine impairs effector T-cell function and promotes the production of Tregs cells, suppressing immunity in the TIME.^101–103^

## Combined TIME-targeted therapy and ICP inhibitor treatment

**Fig. 4:**
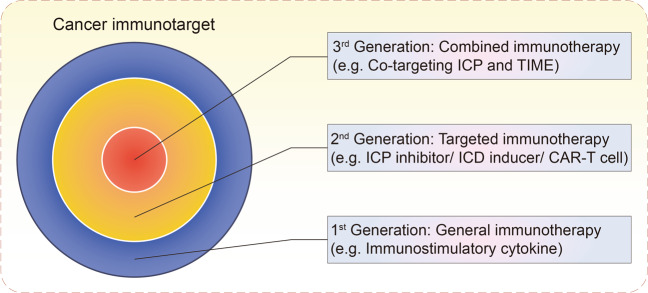
Development and progression of cancer immunotherapeutic strategies. The first-generation of cancer immunotherapy, including but not limited to immunostimulatory cytokines, aimed to generally activate the immune system, so as to promote a concomitant antitumor response. The second-generation of cancer immunotherapy, including but not limited to ICP inhibitors, ICD inducers and CAR-T cells, aimed to block specific immunosuppressive molecules, induce specific cellular processes, or target-specific tumor cells, so as to cause a relatively manageable antitumor response. The third generation of cancer immunotherapy, including but not limited to the co-targeting of ICP and TIME, aimed to jointly inhibit multiple aspects of negative immune regulation, so as to mount an effective and safe antitumor response

There is increasing evidence that demonstrates that the TIME plays a critical role in tumor immune surveillance and immunological evasion.^13–15^ Of the many multiple factors that contribute to anti-cancer immunity, ICPs are just one weapon utilized by tumors to counter attacks from the immune system.^16^ In contrast, as the battlefield on which the tumor and immune system meet, the TIME has an inestimable influence on the final outcome of cancer immunotherapy. Therefore, combined ICP inhibitors (ICIs) and TIME-targeting therapies is a logical strategy to maximize stimulation of an antitumor immune response (Fig. 4).

### Neoantigen-based therapy

Accumulating evidence demonstrates that mutational burden is associated with enhanced antitumor immune response. Therefore, oncologists have attempted to stimulate a neoantigen-specific T-cell response using a variety of approaches. Previous studies have shown that vaccination with a neoantigen incorporating mutant epitopes identified through genomics and bioinformatics induces tumor rejection in a mouse model, providing a theoretical foundation for further neoantigen-based treatment strategies.^104–106^ In general, there are three principal strategies for neoantigen-based therapy. In the first treatment strategy, after identification through genomic sequencing, the neo-peptide most likely to induce the strongest immune response is delivered to patients with or without optimal immune adjuvants.^107,108^ In glioblastoma, the immunogenic antigen was reported to elicit a sustained central memory T-cell response and increased effector T-cell infiltration.^108,109^ Furthermore, complete tumor regression was achieved in melanoma patients through the combination of neoantigen-based therapy with ICI.^110^ The second treatment strategy uses an messenger RNA (mRNA) vaccine. While completely avoiding any possible unwanted modification of patient cells, the mRNA demonstrated satisfactory treatment outcomes in melanoma.^111^ When combined with ICI, a third of patients mounted a complete response after vaccination, resulting in sustained progression-free survival.^111^ The third neoantigen-based therapy focuses on DCs. Firstly, DCs are extracted from the patient and co-incubated with personalized neo-peptides and the corresponding cytokines. The activated DCs are then expanded in vitro and injected back into the patient. The first-generation dendritic vaccination without in vitro stimulation resulted in poor clinical outcomes, with a 3% tumor response rate.^112^ The second-generation dendritic vaccination, which included additional maturation of the DCs in vitro, resulted in an increased response rate of 8–15%.^112^ In 2010, the FDA approved Sipuleucel-T as the sole DC-vaccination therapy for prostate cancer.^113–115^ In a multicenter phase III trial, Sipuleucel-T treatment resulted in a 4.1-month improvement in the median survival in metastatic castration-resistant prostate cancer.^113,116^ The preclinical data also demonstrated that the DC-vaccination therapy combined with ICIs improved CD8^+^ T-cell infiltration of the tumor and survival in vivo.^117–119^ In addition, a retrospective study indicated that in stage III melanoma patients, a considerable number of those who experienced disease progression after DC-vaccination therapy responded to CTLA-4-targeting by use of ipilimumab.^120^ In a phase II study, the response rate was 38% in stage III/IV melanoma patients following administration of ipilimumab combined with DC-vaccination therapy.^121^ These results suggest that the combination of ICIs with DC-vaccination therapy has a promising future.

### Immunogenic cell death induction

Over the past 10 years, mounting evidence has suggested that inducing cancer cell death to activate the immune system is an effective method of anti-cancer immunotherapy.^122–124^ It is well-known that different stimuli activate multiple pathways of cancer cell death, such as apoptosis, necroptosis, autophagy, ferroptosis, and pyroptotic cell death.^125–127^ From an immunological perspective, immunogenic cell death (ICD) is the cause of an adaptive immune response elicited by cell-associated antigens released from dead cells.^122,126,128^ It has been verified that the molecular properties of ICD largely overlap with TIME regulators. Exposure of calreticulin (CALR, an “eat me” signal), secretion of adenosine triphosphate (ATP, a “come to me” signal), release of high mobility group box 1 (HMGB1, an “activate you” signal), autocrine production of type I interferon (IFN I, a “stimulate you” signal), and export of annexin A1 (a “find me” signal) from dying cancer cells have been identified as the five key hallmarks of the process of ICD.^129^ Following the initiation of ICD, secreted ATP favors the recruitment and activation of APCs by P2RY2 and P2RX7.^130^ Exported annexin A1 then guides the homing and juxtaposition of APCs to the dying cells by FPR1.^131^ Subsequently, exposed CALR promotes the engulfment of dying cells and antigen uptake by LRP1.^132^ Furthermore, released HMGB1 stimulates the synthesis of pro-inflammatory factors, maturation of APCs and presentation of tumor antigens by TLR4.^133^ Finally, autonomous IFN I increases secretion of CXCL10 and recruitment of T cells to exert antitumor effects.^134,135^ Of note, such hallmarks of ICD can be triggered by multiple cellular stress conditions, including ER stress-induced CALR exposure, autophagy-induced ATP secretion, secondary necrosis-engendered HMGB1 and annexin A1 release, and stimulation of autonomous IFN I production by infectious pathogens.^130,135–141^ Hence, widely applicable inducers of ER stress, autophagy, necroptosis or viral mimicry are now employed as activators of ICD.^142–149^

Increasing preclinical and clinical evidence has revealed that a conventional chemotherapy-induced immune response is principally dependent on the induction of ICD.^142,143,150,151^ Indeed, pharmacological or genetic suppression of ICD effectors greatly diminishes the curative effects of anthracycline-based immunogenic chemotherapy.^133,134,152–157^ The core phenotypes and mechanisms of immunogenic chemotherapy are highly consistent, at least in anthracycline-treated breast cancer, oxaliplatin-treated colorectal cancer, bortezomib-treated multiple myeloma, and imatinib-treated gastrointestinal stromal cancer, in spite of slight differences in tissue-specificity or drug-precise pharmacological action.^131,158–162^ However, since there are also considerable shortcomings associated with the induction of ICD for cancer therapy, the combination of ICD inducers and ICP-targeting drugs may be an optimal counterplan to assist cancer patients in the future.^4,144,163–165^ For instance, ICD is triggered in combination with a number of undesirable immunosuppressive effects, particularly in anthracycline-based immunogenic chemotherapy,^166^ while bona fide immune interventions can, in certain circumstances, improve treatment efficacy to some extent. In addition, to neutralize the immunosuppressive effects and maximize the immunostimulatory function of anti-cancer drugs, the chemotherapeutic drug gemcitabine has been combined with ipilimumab in preclinical models,^167^ and the BRAF inhibitor dabrafenib and the MEK inhibitor trametinib have also been used in combination with various ICP-targeting agents in experimental studies.^168^ More excitingly, the CDK inhibitor dinaciclib has recently been confirmed to induce ICD and enhance anti-PD-1-mediated tumor suppression in an immunocompetent mouse model.^169^ Furthermore, local chemotherapy synergized with CTLA-4 inhibition has been shown to boost an immune response in mice and patients with advanced melanoma.^170^ Several clinical trials have been launched to evaluate the clinical profile of the synergistic response of combination therapy with ICD inducers and ICP blockers (Table 1). This strategy can, at least in principle, mediate direct destruction of a fraction of cancer cells, while stimulating short-term immune clearance of the remainder, while also maintaining long-lasting immune memory to prevent disease recurrence.

**Table 1:** Clinical trials of TIME and ICP co-targeted combination therapies in multiple cancers

TIME-targeting therapy	Effect	Combined ICP inhibitor	Indication	N	Response	Clinical trial	Status
T-VEC	OV	Ipilimumab	Melanoma	198	ORR 39% (T-VEC + Ipi) vs. 18% (Ipi), *P* = 0.002	NCT01740297	Active, not recruiting
T-VEC	OV	Pembrolizumab	Melanoma	21	ORR 48%	NCT02263508	Active, not recruiting
T-VEC	OV	Pembrolizumab	HNSCC	36	ORR 15.6%; disease control rate 40.6%	NCT02626000	Active, not recruiting
HF10	OV	Ipilimumab	Melanoma	46	BORR at 24 weeks 41%; median PFS 19 m; median OS 21.8 m	NCT02272855	Completed
HF10	OV	Nivolumab	Melanoma	7	Major pathological response 0%	NCT03259425	Active, not recruiting
Epacadostat	IDO1 inhibitor	Pembrolizumab	Gastric cancer and esophageal cancer	3	6-month survival rate 33.3%	NCT03196232	Completed
Epacadostat	IDO1 inhibitor	Pembrolizumab	Head and Neck cancer	54	ORR 31.4% (Epa + Pem) vs. 21.1% (Pem)	NCT03358472	Active, not recruiting
Epacadostat	IDO1 inhibitor	Pembrolizumab	Lung cancer	154	ORR 32.5% (Epa + Pem) vs. 39% (Pem)	NCT03322540	Active, not recruiting
Epacadostat	IDO1 inhibitor	Pembrolizumab	RCC	129	ORR 31.3% (Epa + Pem) vs. 29.2% (SoC)	NCT03260894	Active, not recruiting
Epacadostat/platinum-based chemotherapy	IDO1 inhibitor/chemotherapy	Pembrolizumab	Lung cancer	223	ORR 26.4% (Epa + Pem+Chemo) vs. 44.8% (Pem + Chemo)	NCT03322566	Active, not recruiting
Azacitidine/epacadostat	DNA methyltransferase inhibitor/IDO1 inhibitor	Pembrolizumab	Advanced malignancies	70	5.7% ORR	NCT02959437	Completed
Axitinib	VEGFR inhibitor	Pembrolizumab	RCC	861	median PFS 15.1 m (Axi + Pem) vs. 11.0 m (Suntinib), *P* = 0.00012; ORR 59.3% (Axi + Pem) vs. 35.7% (Suntinib), *P* < 0.0001	NCT02853331	Active, not recruiting
Bevacizumab	VEGFR inhibitor	Atezolizumab	RCC	915	median PFS 11.2 m (Bev + Ate) vs. 7.5 m (Sunitinib), *P* = 0.0205	NCT02420821	Active, not recruiting
Bevacizumab/capecitabine	VEGFR inhibitor	Atezolizumab	CRC	133	median PFS 4.37 m (Bev + Ate+Cap) vs. 3.32 m (Bev + Cap), *P* = 0.051; median OS 10.55 m (Bev + Ate+Cap) vs. 10.61 m (Bev + Cap), *P* = 0.40	NCT02873195	Active, not recruiting
Bevacizumab	VEGF inhibitor	Atezolizumab	HCC	356	median PFS 6.8 m (Bev + Ate) and 4.3 m (Sunitinib), *P* < 0.001; 1-year survival rate 67.2% (Bev + Ate) vs. 54.6% (Sunitinib)	NCT03434379	Active, not recruiting
Bevacizumab/carboplatin+ paclitaxel	VEGF inhibitor/chemotherapy	Atezolizumab	NSCLC	692	median PFS 8.3 m (Bev + Chemo+Ate) vs. 6.8 m (Chemo + Bve), *P* < 0.001; median OS 19.2 m (Bev + Chemo+Ate) vs. 14.7 m (Chemo + Bve), *P* = 0.02	NCT02366143	Active, not recruiting
Carboplatin/nab-paclitaxel/pemetrexed	Chemotherapy	Atezolizumab	NSCLC	723	median PFS 7.0 m (Ate + Nab+Pab+Car) vs. 5.5 m (Nab + Pab+Car), *P* < 0.001; median OS 18.6 m (Ate + Nab+Pab+Car) vs. 13.9 m (Nab + Pab+Car), *P* < 0.001	NCT02367781	Active, not recruiting
Nab-paclitaxel	Chemotherapy	Atezolizumab	Triple-negative breast cancer	900	ITT median OS 21.0 m (Ate + Chemo) vs. 18.7 m (Chemo), *P* = 0.078	NCT02425891	Active, not recruiting
Cisplatin/carboplatin/pemetrexed	Chemotherapy	Pembrolizumab	NSCLC	616	median PFS 8.8 m (Pem + Chemo) vs. 4.9 m (Chemo), *P* < 0.00001; ORR 47.6% vs. 18.9%, *P* < 0.0001	NCT02578680	Active, not recruiting
Carboplatin/etoposide	Chemotherapy	Atezolizumab	NSCLC	403	median PFS 5.2 m (Ate + Chemo) vs. 4.3 m (Chemo), *P* = 0.0170; 12.3 m (Ate + Chemo) vs. 10.3 m (Chemo), *P* = 0.0069	NCT02763579	Active, not recruiting
Carboplatin/nab-paclitaxel/pemetrexed	Chemotherapy	Pembrolizumab	NSCLC	559	median PFS 6.4 m (Pem + Chemo) vs. 4.8 m (Chemo), *P* < 0.0001; OS 15.9 m (Pem + Chemo) vs. 11.3 m (Chemo), *P* = 0.0008; ORR 57.9% (Pem + Chemo) vs. 38.4% (Chemo)	NCT02775435	Active, not recruiting
NKTR-214	IL-2 therapy	Nivolumab/Ipilimumab	Melanoma, NSCLC	38	ORR 59.5%; DCR 83.8%	NCT02983045	Active, not recruiting
M7824	Bifunctional fusion protein composed of a mAb against PD-L1 fused to a TGFβ	/	Solid Tumors	19	ORR 15.7%	NCT02517398	Active, not recruiting

*HNSCC* Head and neck squamous cell carcinoma, *OV* oncolytic virus, *ORR* objective response rate, *DCR* disease control rate, *RCC* renal cell carcinoma, *SoC* stand of care, *PFS* progression-free survival, *HCC* hepatocellular carcinoma, *ITT* intention-to-treat patients, *NSCLC* non-small-cell lung cancer, *CRC* colorectal carcinoma, *vs*. versus, *N* patient number

### Oncolytic viruses

Over recent years, oncolytic viruses (OVs) have attracted significant attention as a promising antitumor therapy based on their capacity for preferential replication in tumor cells, causing lysis and thus transforming a cold immunosuppressive tumor into one which is inflamed.^171,172^ OVs promote an antitumor immune response via a variety of mechanisms. Firstly, direct killing of tumoral cells through activation of different cytocidal programs, including apoptosis, autophagy, necroptosis, and pyroptosis leads to dissemination of a wide repertoire of both progeny virions and cellular tumor-associated antigens/neoantigens into the microenvironment.^171–173^ In addition, lysed tumor cells disseminate additional danger-associated molecular patterns and viral pathogen-associated molecular patterns (PAMPs), which induce an inflammatory immune response.^171–173^ The PAMPs consist of viral RNA, DNA, or proteins that are sensed by pattern recognition receptors expressed by DCs. As a result of pattern recognition receptor engagement, activated DCs produce cytokines that are pro-inflammatory (e.g., TNF-α and IL-12) and antiviral (IFN I: IFN-α and IFN-β), all of which contribute to tumor antigen cross-presentation and cytotoxic T-cell priming.^171–173^ Furthermore, OV-mediated cancer cell death is characterized by the exposure and/or release of danger-associated molecular patterns, including ATP, HMGB1, and CALR, which represent the classical hallmarks of ICD.^171–174^ Last but not least, the sensing of PAMPs by tumor cells triggers an IFN I response, which eventually promotes both NK cell and cytotoxic T-cell antitumor responses.^172,173,175^ In 2015, T-VEC was approved by the FDA as the first oncolytic virus for advanced melanoma therapy.^176^ In a phase III randomized clinical trial (OPTiM) conducted in 436 patients with stage IIIB to IV melanoma, the response rate was significantly higher in those with T-VEC than subcutaneous administration of granulocyte-monocyte colony-stimulating factor alone, demonstrating the capability of T-VEC to boost an antitumor immune response.^177^ Based on these results, a clinical trial combining T-VEC and ipilimumab was conducted in 198 patients with advanced melanoma.^178^ This approach resulted in a significantly increased response rate and prolonged survival compared with the administration of ipilimumab alone.^178^ The combination of ICIs and OVs represents a rational design methodology for immunotherapy. As immunologically “cold” tumors with low mutational burden and inhibited immune cell infiltration may not respond to ICIs, direct oncolysis mediated by armed virotherapy will generate tumor antigens, danger-associated molecular patterns, and PAMPs to prime a T-cell response and reshape the TIME. Thus, this approach has the potential to overcome the primary or adaptive resistance to ICI monotherapies experienced in the clinic (Table 1).

### Cytokine therapy

Cytokines released by various cell types in the TIME in response to cellular stress conditions play a critical role in modulating the influx and expansion of leukocytes. The secreted cytokines enable the rapid propagation of immune signaling in a complex yet efficient manner, and thus can generate a potent and coordinated immune response to target antigens.^179^ The growing interest in harnessing cytokines in the TIME to boost antitumor immunity has been accompanied by intensified efforts to characterize cytokines and exploit their vast signaling networks to develop cancer treatments.^179,180^ As a result, numerous clinical trials have been conducted to investigate the potential antitumor activity of a number of recombinant cytokines. IL-2 was approved as the first immunotherapy for patients with advanced renal cell carcinoma and metastatic melanoma,^181^ followed by the approval of IFN-α for renal cell leukemia, non-Hodgkin lymphoma and melanoma.^179,180^ However, these cytokine strategies failed to live up to expectations raised by the results obtained in preclinical models, with clinical investigations revealing the narrow therapeutic windows and modest antitumor efficacy of such treatments, thus limiting their clinical application as a monotherapy for cancer patients.^172,174,182^ Second-generation, IL-2-based compounds developed with improved pharmacodynamic properties have displayed promising response rates when combined with ICP therapy.^183,184^ In a single-arm, phase I dose-escalation trial, bempegaldesleukin, a CD122-preferential IL-2 pathway agonist, was evaluated in combination with nivolumab in 38 patients with advanced solid tumors (melanoma, renal cell carcinoma, and NSCLC). The total objective response rate was 59.5% (22/37), with seven complete responses (18.9%), in patients with poor prognostic risk factors for response to PD-1/PD-L1 blockade, demonstrating promising therapeutic potential of this novel generation of IL-2 targeted therapy (Table 1).^184^

IL-12 is mainly produced by activated antigen-presenting cells bridging the innate and adaptive immune systems. In preclinical models, the combination of IL-12 and chemotherapy eliminated intratumoral Treg cells and induced the appearance of inflammatory myeloid cells in xenograft mouse models.^185^ However, in early clinical trials, because of the short half-life of the recombinant protein, high and multiple doses were usually required, which induced dose-related adverse events.^186^ In a phase II clinical trial, electroporation of Intratumoral Tavo (plasmid encoding IL-12) was well tolerated and demonstrated strong antitumor activity, which led to a systemic immune response in advanced melanoma patients.^187^ Granulocyte-macrophage colony-stimulating factor (GM-CSF) is a cytokine that promotes the expansion and activation of dendritic cells for antigen presentation, and activates T- and B-lymphocyte functions. In phase 3 adjuvant trials for melanoma and lymphoma, systemic administration of GM-CSF demonstrated antitumor effects.^188^ However, previous reports have also shown that GM-CSF may have negative effects on an immune response as it may induce accumulation of MDSCs, which promote tumor growth. In a phase III clinical trial, the ipilimumab plus sargramostim (GM-SCF) group achieved superior overall and progression-free survival outcomes than ipilimumab alone in patients with metastatic melanoma.^189^ Generally, cytokines are promising and complex TIME targets. The cytokine-based therapy may help overcome the primary and acquired resistance of ICBs and maximize the clinical benefit in a wide range of patients due to its capacity to promote immune cell infiltration and activation of the lymphocyte fraction.

### Antiangiogenic therapy

As a hallmark of TIME in a variety of malignancies, the vascular abnormalities of tumors are significantly correlated with immune suppression and tumor cell evasion. Vascular endothelial growth factor (VEGF) and angiopoietin (ANG2) play important roles in regulating tumor angiogenesis. Firstly, VEGF promotes immunosuppression as poor perfusion results in abnormal vascular restricted drug delivery and immune cells infiltration. Moreover, hypoxia and the low pH tumor microenvironment modulate the activity of immune cells. In addition, VEGF and ANG2 can also alter the expression levels of adhesion molecules on endothelial cells that impair the trafficking of immune cells from vessels to TIME.^190^ Furthermore, increased VEGF and ANG2 also result in the recruitment and proliferation of immunosuppressive cells, including but not limited to Treg cells, MDSCs, and TAMs.

Antiangiogenic therapy is among the most promising treatments for a variety of malignancies due to its ability to induce durable tumor regression by starving the tumor of blood and nutrient supplies. In addition to its ability to suppress sprouting angiogenesis and delay tumor growth, previous studies have shown that administration of antiangiogenic drugs also leads to tumoral vascular normalization, including upregulation of the leukocyte adhesion molecules (intercellular adhesion molecule 1 and vascular cell adhesion molecule 1), as well as enhancement of blood perfusion and oxygen levels in the TIME. These changes result in increased T-cell infiltration and eventually convert an immunosuppressive microenvironment into one, which is immunosupportive.^191–193^ Therefore, a number of scientists have suggested that the use of ICBs during the window of “vascular normalization” may result in superior clinical outcomes. In a preclinical breast cancer model, targeting the tumor vasculature resulted in a more homogeneous distribution of functional tumor vessels.^194^ In addition, administration of a low dose of an antibody targeting VEGFR2 converted tumor-associated macrophages from an immune suppressive M2-like phenotype toward an immune stimulatory M1-like phenotype that can boost CD4^+^ and CD8^+^ T-cell tumor infiltration.^194^ Moreover, preclinical data indicated that immunotherapy combined with anti-VEGF antibody resulted in a significant increase in treatment efficacy compared with immunotherapy alone.^191,195^ The results of a phase III trial demonstrated the benefit of such combination treatment for patients with advanced-stage renal cell carcinoma (RCC).^196^ In HCC, a phase III trial showed that atezolizumab combined with bevacizumab resulted in better overall and progression-free survival outcomes than sorafenib.^197^ In other clinic trials, combining antiangiogenic therapy with ICIs has displayed promising antitumor activity with an acceptable safety profile in patients with gastric cancer, melanoma, and non-small-cell lung cancer (Table 1).^175,198–201^ These results from both preclinical and retrospective clinical studies have demonstrated that vascular normalization induced by targeting the VEGF pathway improved the effectiveness of ICBs. However, over time, a tumor may again switch back to a hypoxic environment and escape from this combination therapy. The underlying mechanism remains incompletely understood and there is still an urgent need to optimize the dosage, duration, and sequence of administration of ICBs and antiangiogenic agents for this combination treatment.

### Targeting CAFs and the extracellular matrix

CAFs and the extracellular matrix play critical roles in the development and maintenance of an immunosuppressive microenvironment. Firstly, CAFs have been shown to recruit Treg cells, MDSCs and TAMs into tumor environment in varying malignancies, which promote the formation of an immunosuppressive milieu.^202–204^ In addition, accumulating evidence also suggests that CAFs may reprogram infiltrated immune cells toward a tumor-promoting function^205,206^ (Table 2). Moreover, the excessive deposition of collagen due to activation of CAFs may result in formation of scar-like tissue, especially in pancreatic cancer, which may be utilized by tumor cells as a physical barrier to prevent T-cell infiltration into a tumor.^207^ Therefore, co-targeting CAFs may improve the efficacy of immunotherapy due to its role in TIME. However, depilation of either FAP^+^ or α-SMA^+^ cell populations has resulted in severe systematic problems in preclinical models, suggesting that these fibroblasts have important physical function as both FAP and α-SMA are not expressed exclusively in CAFs. Therefore, targeting the signaling pathway critical for CAF activation may be more realistic. The TGFβ signaling pathway has been shown to play an important role in the proliferation and activation of CAFs and the suppression of CD8^+^ T-cell recruitment to the tumor in addition to inhibition of their function.^208^ Inhibition of the TGFβ signaling pathway in CAFs has been found to result in reduced collagen I secretion, resulting in reduced extracellular matrix stiffness.^209^ Previous studies have shown that tumor resistance to ICP-targeting therapy was associated with a TGFβ signaling signature in fibroblasts.^208,210^ Combining TGFβ-blockade with anti-PD-L1 antibodies has reduced TGFβ signaling in stromal cells, facilitated T-cell infiltration, and provoked vigorous antitumor immunity and tumor regression in a mouse model.^210^ Fibroblast activation protein-positive CAFs are the primary source of CXCL12 in pancreatic cancer. Combining CXCL12-targeted therapy with anti-PD-L1 immunotherapy was shown to result in a synergistic effect in pancreatic cancer.^211^ Effective stromal modulation was also demonstrated by the administration of a polymeric micelle-based nano-formulation encoding liver Hedgehog inhibitor and a chemotherapy drug (M-CPA/PTX). This combination modulated the pancreatic cancer stroma by increasing intratumoral vascular density. When ICP blockade and an anti-PD-1 antibody was included in this combination, it promoted tumor infiltration of cytotoxic CD8^+^ T cells that prolonged survival in a mouse model of pancreatic cancer.^212^ Taken together, CAF has become an attractive target for combination immunotherapy due to its essential role in mediating TIME. However, current targeting therapy seems a double-edged sword as neither these targeted molecules nor cytokines can be exclusively attributed to CAFs. Highly advanced nanoparticle delivering systems or other approaches may help solve these problems, which only the future will reveal.

**Table 2:** CAF-reprogrammed effects on immune cells

CAF signal molecules	Immune cell types	Regulatory effects
CXCL12, CAF-S1, OX40L, PD-L2, JAM2	CD4^+^ CD25^+^ T cells	Recruitment, Treg differentiation
SDF-1, IL-6, CCL5, CCL2, SDF-1	Circulating monocytes	Recruitment, M2-like differentiation
SDF-1, DKK1, MCP-1	Peripheral blood neutrophils	Recruitment, MDSCs differentiation, activation
PGE2	NK cells	Inhibition of NK cytotoxic activity
CXCL12, TGF-β, PD-L1/2, COX-2, FAS-L	T cells	Exclusion, suppression of proliferation and activation
IL-6, DPP4	Helper T cells	Inhibition of Th1, Th17 differentiation

*CXCL12* C-X-C motif chemokine ligand 12, *OX40L* oxford 40 ligand, *JAM2* junction adhesion molecule 2, *SDF-1* stromal cell-derived factor 1, *IL-6* interleukin-6, *CCL5* C-C motif chemokine ligand 5, *DKK1* dickkopf WNT signaling pathway inhibitor 1, *MCP-1* monocyte chemoattractant protein 1, *PGE2* prostaglandin E2, *MDSC* myeloid-derived suppressor cell

## Conclusions

In conclusion, TIME regulators are promising cancer immunotherapeutic targets, particularly for solid tumors. Of note, TIME is an interesting but largely unexploited field. So far, there is less clinical evidence for a TIME-targeted approach compared with antibodies against PD-1/PD-L1 or CTLA-4. Many studies of the TIME are based on preclinical mouse models or quite small patient cohorts. Studies of larger patient cohorts have mostly investigated melanoma. In other words, the presented literature does not sufficiently establish that the TIME is the major explanation for the failure of cancer immunotherapy in humans or that new drugs modulating TIME could overcome this limitation. To further solve the complexity of TIME (including the heterogeneity of tumors), investigations across different patient populations and cancer types at both single cellular and molecular levels may provide practicable and precise solutions for wider clinical application, particularly for improvements in the therapeutic efficacy of targeting TIME regulators in addition to its combination with immune checkpoint inhibitors.
